# Dapagliflozin Reduces Kidney Inflammation in Alport Syndrome by Inhibiting the Stimulator of IFN Genes Pathway in Renal Tubular Epithelial Cells

**DOI:** 10.34067/KID.0000001099

**Published:** 2026-01-07

**Authors:** Qimin Zheng, Yafei Zhao, Yuanmeng Jin, Shuwen Yu, Yunzi Liu, Hanlan Yu, Zhengying Fang, Li Yang, Qinjie Weng, Jing Xu, Xiaoxia Pan, Xiangchen Gu, Jingyuan Xie

**Affiliations:** 1 Department of Nephrology, Shanghai Ruijin Hospital, School of Medicine, Shanghai Jiao Tong University, Shanghai, China; 2 Institute of Nephrology, School of Medicine, Shanghai Jiao Tong University, Shanghai, China; 3 Department of Nephrology, Yueyang Hospital of Integrated Traditional Chinese and Western Medicine, Shanghai University of Traditional Chinese Medicine, Shanghai, China

**Keywords:** Alport syndrome, chronic inflammation, renal tubular epithelial cells, SGLT2 inhibitors

## Abstract

**Key Points:**

- Dapagliflozin reduces proteinuria in patients with Alport syndrome.
- Dapagliflozin plays an anti-inflammatory role by inhibiting the stimulator of IFN genes pathway in tubular epithelial cells of Alport syndrome mice.

**Background:**

Alport syndrome (AS) is a hereditary kidney disease caused by *COL4A3/4/5* mutations that lack of effective treatments. Sodium-glucose cotransporter 2 inhibitors have demonstrated renal and cardiovascular protective effects in patients with CKD; however, their long-term effects in patients with AS and the underlying mechanisms remain to be clarified.

**Methods:**

We conducted a single-arm, prospective study to examine the effect of dapagliflozin in patients with AS. In parallel, *Col4a3* p.C1615Y-mutant mice (129S2/Sv background) were used as an AS model to investigate the renoprotective mechanisms of dapagliflozin.

**Results:**

A total of twenty-one patients with AS were enrolled. After approximately 12 months of follow-up (12.6±1.2 months), the mean 24-hour urinary protein decreased by 29% to 1.25±0.73 g from baseline (1.75±0.90; *P* < 0.001). The eGFR showed no significant difference compared with baseline (76±28 versus 77±29 ml/min per 1.73 m^2^, *P* = 0.57). In the animal studies, dapagliflozin significantly reduced macrophage infiltration and the expression of inflammatory cytokines levels in the renal cortex of *Col4a3*-mutant mice. Mechanistic studies showed that stimulator of IFN genes (STING) pathway was activated in the renal cortex and tubular epithelial cells (TECs) from *Col4a3* mice, contributing to a proinflammatory phenotype. Dapagliflozin effectively inhibited STING activation and suppressed inflammatory cytokines production in mutant TECs.

**Conclusions:**

Dapagliflozin can reduce proteinuria in patients with AS and plays an anti-inflammatory role by inhibiting the STING pathway in TECs of AS mice.

## Visual Abstract

**Figure:**
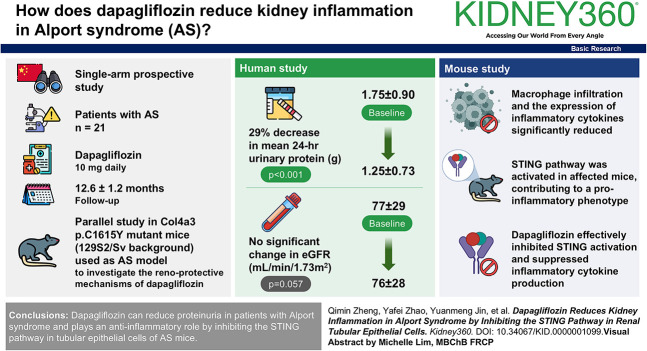

## Introduction

Alport syndrome (AS) is a heredity disease caused by pathogenic mutations in *COL4A3/4/5* and affects multiple organs. Renal manifestations include hematuria, proteinuria, and progressive renal failure.^1^ A study performed in 2021 reported a frequency for pathogenic variants in *COL4A5* of 1/2320, while the frequencies of *COL4A3* and *COL4A4* reached 1/106, suggesting AS prevalence is much higher than previously recognized.^2^ Currently, renin-angiotensin system inhibitors are the only standard therapy for AS.^3^ However, the clinical efficacy remains limited, underscoring the urgent need for novel treatment strategies.

Sodium-glucose cotransporter 2 inhibitors (SGLT2i) were initially developed as antihyperglycemic agent, which lowers blood glucose by promoting urinary glucose excretion.^4^ A landmark study of patients with CKD, A Study to Evaluate the Effect of Dapagliflozin on Renal Outcomes and Cardiovascular Mortality in Patients With CKD (DAPA-CKD), reported a 39% reduction in the renal composite end point in the dapagliflozin group compared with placebo group, suggesting that SGLT2i delays the decline of renal function in patients with CKD.^5^ Some clinical studies have suggested that SGLT2i may reduce proteinuria in AS,^6–9^ but the sample sizes have been limited, and the follow-up is relatively short. The efficacy of dapagliflozin in patients with AS patients requires further clinical data for validation.

The renoprotective effect of SGLT2i has classically been attributed to a tubuloglomerular feedback, leading to afferent arteriolar vasoconstriction and reduced intraglomerular pressure. More recently, SGLT2i has been shown to exert anti-inflammatory effects, mitigating oxidative stress, metabolism dysregulation, impaired autophagy, and inflammation in podocytes and tubular epithelial cells (TECs).^10–12^ Persistent activation of proinflammatory pathways and infiltration of immune cells are recognized drivers of CKD progression, including AS.^13–15^ The cyclic-GMP-AMP synthase-stimulator of IFN genes pathway, a central regulator of inflammation, has drawn increasing attention in CKD.^16^ Whether stimulator of IFN genes (STING) contributes to renal inflammation in AS, however, remains unknown.

Although much of AS pathogenesis has traditionally been attributed to podocyte injury, emerging evidence highlights a critical role for tubular cells in AS. First, the COL4A3/4/5 trimer contributes to distal tubular basement membrane,^17^ and single-cell RNA sequencing demonstrates that *COL4A3* is expressed in renal TECs.^18,19^ Histopathologic findings in AS, including tubular vacuolar degeneration, interstitial inflammation, and foam cell infiltration, further implicate tubular injury.^20,21^ In addition, tubular inflammation and metabolic disorders have been reported in AS-related kidney damage progression.^14,22^ Despite the well-recognized tubular protection conferred by SGLT2i in CKD, their role in AS tubulopathy has not been explored.

In this study, we evaluated the clinical efficacy of dapagliflozin in patients with AS and further investigated its mechanisms in *Col4a3* p.C1615Y-mutant mice, focusing particularly on renal tubular injury and inflammation.

## Methods

### Patients

A single-arm, prospective study enrolled patients with AS in Ruijin Hospital from May 2021 to January 2025. Inclusion criteria were (*1*) age ≥18 years; (*2*) diagnosis of AS based on clinical, pathologic, and genetic findings; (*3*) stable dose of angiotensin-converting enzyme inhibitor or angiotensin II receptor blocker for at least 3 months; and (*4*) follow-up of more than 12 months. Exclusion criteria were (*1*) comorbid type 2 diabetes and (*2*) use of steroids or immunosuppressive therapy. Patients were administered dapagliflozin (10 mg once daily), and urinary protein, serum creatinine (Scr), and adverse events were recorded at each follow-up visit. The study was approved by the Ethics Committee of Shanghai Ruijin Hospital (Approval No. “2016伦理第15号”). This study adheres to the Declaration of Helsinki, and informed consent was obtained from all participants.

### Animal Experiments

The animal model established with the CRISPR/Cas9 technology was *Col4a3* p.C1615Y transgenic mice in a 129S2/Sv background. This missense mutation corresponds to amino acid number 1616 in humans and was first identified in a large AS family who underwent renal biopsy and genetic testing in our department.^23^ Exon sequencing of the *Col4a3* gene and Sanger sequencing were performed to confirm the genotype. Mice were housed in a specific pathogen-free facility under a 12-hour light-dark cycle with free access to food and water. To avoid potential disease heterogeneity because of sex differences, *Col4a3* p.C1615Y homozygous-mutant male mice and their wild-type male littermates aged 17 weeks were randomly divided into two groups (experimental group and control group) by number, respectively. The experimental group administered 10 mg/kg per day dapagliflozin and the control group with equivalent vehicle (dimethylsulfoxide) in drinking water for 28 days (wild-type [WT]+vehicle [*n*=5], WT+dapagliflozin [Dapa] [*n*=4], mutant [MUT]+vehicle [*n*=5], MUT+Dapa [*n*=5]). All animal experiments were approved by the Animal Care Committee of Shanghai Jiao Tong University School of Medicine.

### Physiologic and Biochemical Parameters

Mouse urine was collected in metabolic cages on day 29. Urinary albumin was measured with a Mouse Albumin ELISA Kit (ab108792), and urine creatinine was determined with a QuantiChromTM Creatinine Assay Kit (BioAssay Systems, DICT-500). Urinary albumin to creatinine ratio (ACR) was determined to evaluate proteinuria. Mouse blood was collected from the orbital vein under anesthesia on day 30 and then centrifuged at 5000 rpm for 10 minutes to collect serum. Scr and BUN were evaluated with QuantiChromTM Creatinine Assay Kit (BioAssay Systems, DICT-500) and Urea Nitrogen Colorimetric Detection Kit (BioAssay Systems, K024-H1), respectively, according to the manufacturer's instructions.

### Histologic Analysis

Fresh mouse kidney tissues were fixed with 4% paraformaldehyde and paraffin embedded, followed by hematoxylin and eosin (H&E) staining using standard protocols.

### Immunofluorescence

After deparaffinization, antigen retrieval, and blocking, kidney sections were incubated overnight at 4°C with rabbit anti-STING primary antibodies (Proteintech #19851-1-AP). Sections washed with PBS were incubated with Alexa Fluor594 donkey anti-rabbit lgG (H+L) secondary antibodies at 37°C in the dark for 1 hour. After rinsing again with PBS, a 4′,6-diamidino-2-phenylindole working solution was added dropwise to each section. Nuclei were stained for 5 minutes away from light and rinsed with PBS.

### Immunohistochemistry

Kidneys were fixed with 4% paraformaldehyde, dehydrated, paraffin embedded, and cut into 4-*μ*m sections. The sections were then incubated with hydrogen peroxide solution to block endogenous peroxidase and then blocked with BSA for 30 minutes. The sections were next incubated with anti-F4/80 (Cell Signaling Technology#70076) primary antibodies at 4°C overnight. After incubation with horseradish peroxidase-conjugated secondary antibodies for 30 minutes at room temperature, the slides were rinsed again and stained with a 3,3′-diaminobenzidine Staining Kit.

### Primary Culture of Mouse TECs

As previously described,^24^ kidneys were collected from 4 to 6 week-old mice, minced, and digested with 2 mg/ml collagenase IV for 40 minutes at 37°C and then filtered successively through 100-, 70-, and 40-*μ*m mesh to collect TECs. Cells were cultured in Roswell Park Memorial Institute 1640 medium supplemented with 10% FBS, 20 ng/ml EGF, 1% penicillin/streptomycin and 1× insulin–transferrin–selenium with 5% CO_2_ at 37°C. At 80% confluence, cells were starved in 0.5% FBS (Roswell Park Memorial Institute) overnight and administered the proper treatment for 24 hours, including 20 *µ*M dapagliflozin (AstraZeneca), 10 ng/ml TNF-*α* (R&D #210-TA-020), 100 ng/ml LPS (BIOAGRIO, Shanghai, China), 3 *µ*M STING inhibitor (MedChemExpress #HY-138682), and 30 *µ*M STING agonist (MedChemExpress #HY-12885B).

### Quantitative Real-Time PCR

Total RNA was isolated from cells and tissues with an RNA Isolation Kit (Vazyme, Nanjing, China) according to the manufacturer's instructions. RNA was reverse transcribed with a High-Capacity cDNA Reverse Transcription kit (Vazyme). qPCR was performed with the SYBR Green PCR Master Mix (Vazyme) on a StepOne Plus Real-Time PCR System (Applied Biosystems; Thermo Fisher Scientific). The used primers are listed in Supplemental Table 1.

### Renal Cortex RNA-Seq Analysis

Mouse kidney cortex samples from four groups (WT+vehicle, WT+dapa, MUT+vehicle, MUT+dapa) were collected for RNA-seq. Three samples were randomly selected from each group. Total RNA was extracted with TRIzol reagent (Invitrogen, CA) according to the manufacturer's instructions. Then libraries were constructed with the VAHTS Universal V6 RNA-seq Library Prep Kit according to the manufacturer's instructions. Transcriptome sequencing and analysis were conducted by OE Biotech Co., Ltd. (Shanghai, China).

### Western Blot

Kidney tissues and primary TECs were homogenized with the radio-immunoprecipitation assay buffer containing protease and phosphatase inhibitor cocktails. The samples were centrifuged at 12,000 rpm for 20 minutes at 4°C, and protein concentrations were determined with the bicinchoninic acid protein assay kit (Epizyme, Shanghai, China). Total protein was separated by SDS-PAGE and transferred onto polyvinylidene fluoride membrane. The membranes were next blocked with 1× protein-free rapid sealing Solution for 20 minutes and incubated overnight with primary antibodies at 4°C. After rinsing the membranes with PBS three times, incubation was performed with anti-rabbit IgG horseradish peroxidase-linked antibody (Cell Signaling Technology #7074P2) for 1 hour. Visualization was carried out with an enhanced chemiluminescence advanced kit, followed by detection on an imaging system (Tanon Science & Technology, Shanghai, China). Western blot data were quantified with IMAGE J. Primary antibodies included *β*-ACTIN antibody (Proteintech#81115-1-RR), STING polyclonal antibody (Proteintech#19851-1-AP), phospho-TBK1 antibody (Cell Signaling Technology #5483T), TBK1 polyclonal antibody (Proteintech#28397-1-AP), phospho-NF-κB p65 antibody (Cell Signaling Technology#3033), NF-κB p65 antibody (Cell Signaling Technology#8242), phospho-IFN-regulatory factor 3 (IRF3) antibody (Cell Signaling Technology#4947), and IRF3 recombinant antibody (Proteintech#80519-1-RR).

### ELISA

Soluble monocyte chemoattractant protein-1 (MCP-1) and regulated upon activation, normal *T* cell expressed and secreted (RANTES) were quantified in tissue lysates from mouse kidneys with mouse MCP-1 (Anogen, Beijing, China) and RANTES (Lianke, Hangzhou, China) ELISA kits. Each renal lysate was diluted in reagent diluent and subjected to ELISA according to the manufacturer's instructions.

### Statistical Analysis

Continuous variables with normal distribution were presented as mean±SD and compared between groups by the Student *t* test. Multiple group comparisons were performed by one-way or two-way ANOVA. All data were analyzed with GraphPad Prism 9.0. *P* < 0.05 was considered statistically significant.

## Results

### Dapagliflozin Reduces Proteinuria in Patients With AS

**Figure 1:**
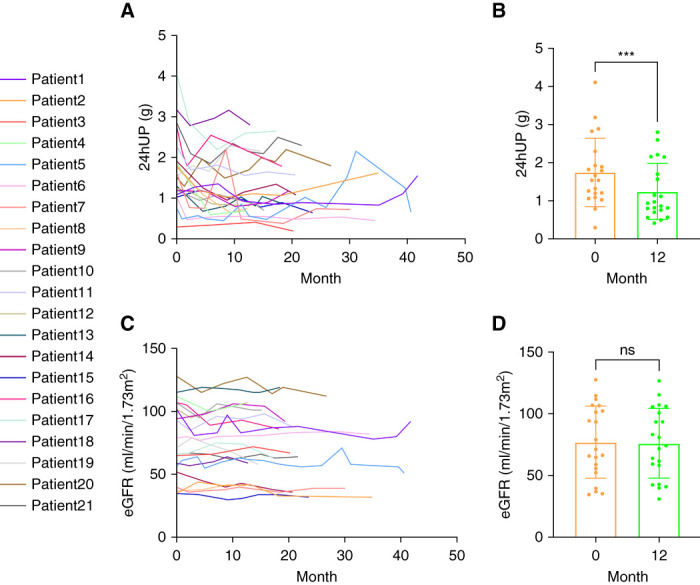
**Changes of urine protein and renal function in patients with AS during dapagliflozin treatment.** (A) Line chart of 24hUP in 21 patients during follow-up. (B) Changes in 24hUP from baseline to 12 months in 21 patients. (C) Line chart of eGFR in 21 patients during follow-up. (D) Changes in eGFR from baseline to 12 months in 21 patients. The baseline and post-treatment parameters were compared using paired *t* test. ***P < 0.001. 24hUP, 24-hour urine protein; AS, Alport syndrome; ns, no significance.

We conducted a single-arm, prospective study to examine the effect of dapagliflozin on patients with AS. A total of 21 patients were enrolled (seven autosomal dominant alport syndrome, two autosomal recessive alport syndrome [ARAS], and 12 X-linked alport syndrome [XLAS]). Their genetic mutation information and clinical characteristics are shown in Table 1. The mean age of patients was 36±13 years, with a mean follow-up period of 22.3±9.1 months. Urine protein and renal function at baseline and the last follow-up for each patient are shown in Supplemental Table 2. The trend of urine protein changes for each patient during the follow-up period is shown in Figure 1A. After approximately 12 months (12.6±1.2) of treatment with dapagliflozin, the patients’ 24-hour urine protein decreased to 1.25±0.73 g, representing a 29% reduction compared with baseline (1.75±0.90; *P* < 0.001; Figure 1B). The trend of eGFR changes for each patient is shown in Figure 1C. After approximately 12 months, the mean eGFR of the patients was 76±28 ml/min per 1.73 m^2^, showing no significant difference compared with baseline (77±29; *P* = 0.57; Figure 1D). No adverse events occurred in these patients during dapagliflozin treatment.

**Table 1:** Genetic mutation information and clinicopathologic characteristic of patients

No	Sex	Age (yr)	Mutation	Genotype	ACMG	Renal Biopsy (LM)	Renal biopsy (EM)	Skin Biopsy
1	F	44	*COL4A4*(p.G1273R)	Heterozygous	LP	MCD	TBMN	—
2	M	60	*COL4A4*(p.G1273R)	Heterozygous	LP	FSGS	Irregular thickness and segmental lamellation of the GBM	—
3	M	49	*COL4A4*(p.K1671X)	Heterozygous	LP	FSGS	Irregular thickness and segmental lamellation of the GBM	—
4	F	33	*COL4A3* (c.4154-2A>G)	Heterozygous	P	FSGS	TBMN	—
5	F	27	*COL4A5* (c.4298-1G>A)	Heterozygous	LP	FSGS	Irregular thickness and segmental split of the GBM	—
6	F	35	*COL4A5*(p.Q645X)	Heterozygous	P	—	—	Mosaic *α*5 (IV) chain staining
7	F	36	*COL4A5* (c.546+4A>T)	Heterozygous	P	FSGS	Irregular thickness, segmental split and basket-weave pattern of the GBM	Mosaic *α*5 (IV) chain staining
8	F	61	*COL4A4*(p.G957R)	Heterozygous	LP	FSGS	Irregular thickness of the GBM	—
9	F	33	COL4A5 (p.R1677Q)	Heterozygous	LP	MCD	TBMN	Mosaic *α*5 (IV) chain staining
10	F	19	COL4A5 (c.1516+2T>C)	Heterozygous	P	FSGS	Irregular thickness and lamellation and basket-weave pattern of the GBM	—
11	F	40	COL4A3 (c.3418+1G>T)	Heterozygous	P	FSGS	Irregular thickness and segmental lamellation of the GBM	—
12	F	28	COL4A5 (p.G1107R)	Heterozygous	P	MCD	TBMN	—
13	F	32	COL4A5 (p.G230V)	Heterozygous	LP	—	—	—
14	F	48	COL4A4 (p.P878fs)	Heterozygous	P	FSGS	Segmental thinning of the GBM	—
15	F	45	COL4A5 (p.G603D)	Heterozygous	LP	FSGS	Segmental thinning of the GBM	—
16	M	20	COL4A4(p.F1517C; p.G484R)	Compound heterozygous	VUS	FSGS	Irregular thickness, lamellation, and basket-weave pattern of the GBM	—
17	M	58	COL4A3(p.P879L; p.R1194S)	Compound heterozygous	VUS	FSGS	Diffuse thinning, segmental split, and lamellation of the GBM	—
18	M	22	COL4A5(p.G527R)	Hemizygous	LP	—	—	—
19	M	24	COL4A5(p.G653A)	Hemizygous	LP	FSGS	Segmental split and basket- weave of the GBM	—
20	M	22	COL4A5(p.G1000V)	Hemizygous	LP	Mild mesangial proliferation	Irregular thickness, segmental split, and lamellation of the GBM	—
21	M	28	COL4A5(c.1424-2A>G)	Hemizygous	LP	FSGS	Global glomerulosclerosis	—

ACGM, American College of Medical Genetics and Genomics; EM, electron microscopy; GBM, glomerular basement membrane; LM, light microscopy; LP, likely pathogenic; MCD, minimal change disease; P, pathogenic; TBMN, thin basement membrane nephropathy; VUS, variant of uncertain significance.

### Dapagliflozin Reduces Proteinuria in *Col4a3*-Mutant Mice

**Figure 2:**
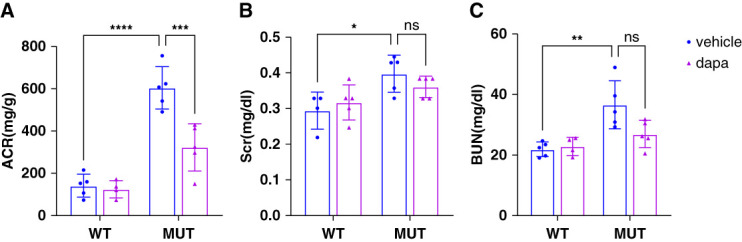
**Dapagliflozin reduces proteinuria in *Col4a3*-mutant mice.** (A) Urinary ACR, (B) Scr and (C) BUN of mice in four groups. *P < 0.05, * * *P* < 0.01, * * *P < 0.001, and * * **P < 0.0001. ACR, albumin-creatinine ratio; dapa, dapagliflozin; MUT, mutant; Scr, serum creatinine; WT, wild-type.

To investigate the protective mechanism of dapagliflozin in AS, *Col4a3* p.C1615Y-mutant mice were used as an animal model of AS, as previously described.^25^ Mutant and wild-type male mice, aged 17 weeks, were randomly divided into two groups (experimental group and control group). The experimental group was administered 10 mg/kg per day dapagliflozin, and the control group received an equivalent volume of vehicle (dimethylsulfoxide) in drinking water for 28 days. Urine samples for urinary ACR detection were collected on day 29, and venous blood and kidney tissue samples were harvested on day 30. The results showed that dapagliflozin significantly reduced ACR in mutant mice (Figure 2A). Meanwhile, renal function reflected by Scr and BUN had a trend of improvement after dapagliflozin treatment (Figure 2, B and C).

### Dapagliflozin Reduces Renal Inflammation in *Col4a3*-Mutant Mice

**Figure 3:**
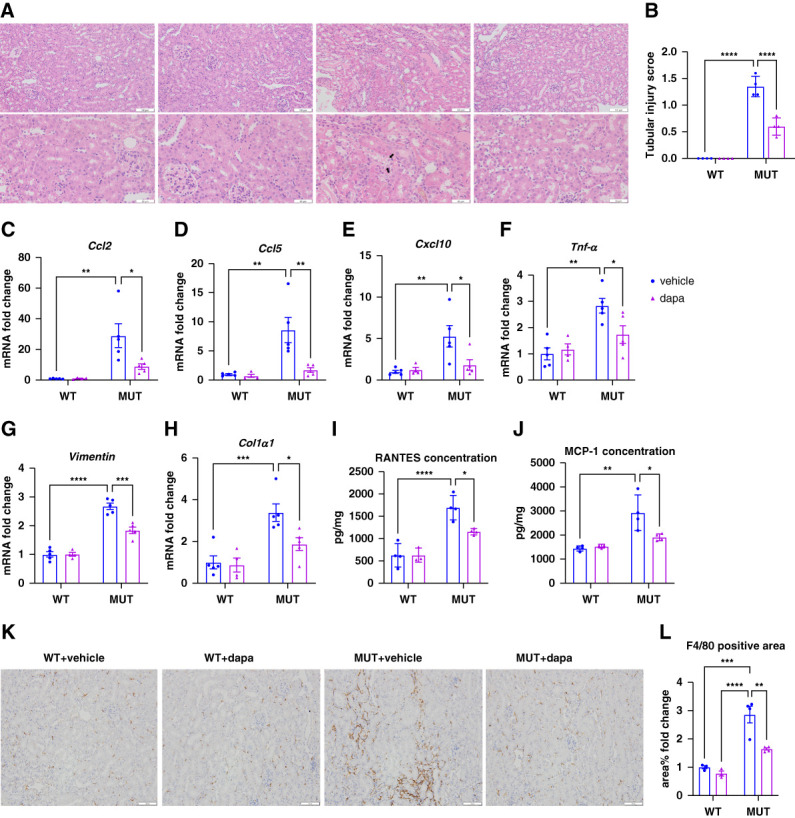
**Dapagliflozin reduces renal inflammation in *Col4a3* mutant mice.** (A) Representative HE staining images of mouse kidneys in each group, bar=100 *μ*m (above), bar=50 *μ*m (below). The black arrows indicate tubular vacuolar degeneration. (B) The bar graph shows the tubular injury score. (C–H) Transcription level of *Ccl2*, *Ccl5*, *Cxcl10*, *Tnf-a*, *Vimentin*, *Col1a1* of kidney cortex in four groups, and *Gapdh* was used as an internal reference. (I and J) RANTES, MCP-1 concentration in the renal cortex of mice in each group; (K) F4/80 staining representative images of mouse kidney in each group, bar: 100 *μ*m. (L) Quantitative of the positive area of F4/80 staining in the kidney of mouse kidney in each group. *P < 0.05, *P < 0.01, ***P < 0.001, ****P < 0.0001. HE, hematoxylin and eosin; MCP-1, monocyte chemoattractant protein-1; RANTES, regulated upon activation, normal T cell expressed and secreted.

Histologic analysis revealed that *Col4a3*-mutant mice developed tubular injuries, including renal tubule lumen dilation and vacuolar degeneration as shown by H&E staining, leading to an increased tubular injury score. These changes were attenuated in mice treated with dapagliflozin (Figure 3, A and B). To determine the key pathways involved, RNA sequencing was performed on the renal cortex in various groups. Kyoto Encyclopedia of Genes and Genomes enrichment analysis of differential expressed genes showed that significant upregulation of immune-related pathways in mutant mice compared with wild-type animals (Supplemental Figure 1A). Notably, dapagliflozin treatment decreased immune pathway activation (Supplemental Figure 1B). Renal cortex qPCR analysis further confirmed that increased transcript levels of proinflammatory cytokines (*Ccl2*, *Ccl5*, *Cxcl10*, *Tnf-a*) and profibrotic genes *(Vimentin* and *Col1a1)* were significantly increased in mutant mice, which were markedly decreased after dapagliflozin treatment (Figure 3, C–H). In line with transcription levels, ELISA showed significantly higher MCP-1 (encoded by *Ccl2*) and RANTES (encoded by *Ccl5*) protein levels in the renal cortex of mutant mice, which were reduced significantly by dapagliflozin (Figure 3, I and J). Immunostaining for F4/80 revealed increased macrophage infiltration in mutant kidneys that was also alleviated with dapagliflozin (Figure 3, K and L). Together, these data confirmed that *Col4a3*-mutant mice exhibit robust immune activation and inflammation, which are significantly attenuated by dapagliflozin.

### Dapagliflozin Attenuates the Proinflammatory Phenotype in TECs of *Col4a3*-Mutant Mice

**Figure 4:**
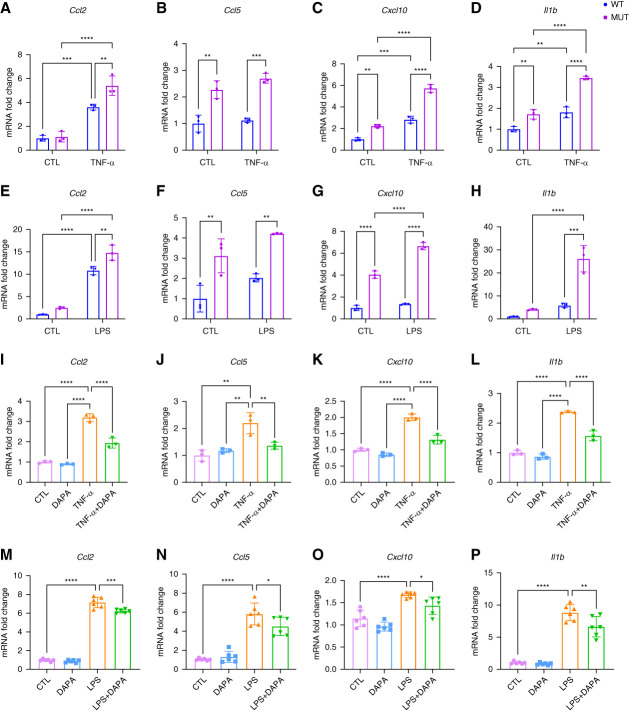
**Dapagliflozin attenuates proinflammation phenotype in proximal TECs of *Col4a3*-mutant mice.** (A–D) Transcription level of *Ccl2*, *Ccl5*, *Cxcl10*, and *Il1b* in four groups of TECs. (E–H) Transcription level of *Ccl2*, *Ccl5*, *Cxcl10*, and *Il1b* in four groups of TECs. (I–L) Transcription level of *Ccl2*, *Ccl5*, *Cxcl10*, and *Il1b* in mutant TECs under different stimulation. (M–P) Transcription level of *Ccl2*, *Ccl5*, *Cxcl10*, and *Il1b* in mutant TECs under different stimulation. *P < 0.05, * *P* < 0.01,*** *P* < 0.001,**** *P* < 0.0001. CTL, control; TECs, tubular epithelial cells.

Renal TECs are key intrinsic cells that secrete proinflammatory cytokines and chemokines. To examine whether *Col4a3*-mutant TECs display heightened inflammatory susceptibility, primary wild-type and mutant proximal TECs were treated with TNF-*α*. Mutant TECs exhibited significantly elevated baseline transcript levels of *Ccl2*, *Cxcl10*, and *Il1b* transcript levels, which were further increased following TNF-*α* stimulation. *Ccl5* transcription showed a tendency to increase in mutant TECs compared with wild-type cells after TNF-*α* exposure (Figure 4, A–D). To validate these findings, TECs were further stimulated with LPS. When stimulated with LPS, mutant TECs again exhibited elevated levels of *Ccl2*, *Cxcl10*, and *Il1b*, along with an increasing trend in *Ccl5* mRNA levels compared with wild-type controls (Figure 4, E–H). Then, treatment of mutant TECs with dapagliflozin suppressed TNF-*α*–induced upregulation of these cytokines (Figure 4, I–L) and also inhibited LPS induced cytokine expression (Figure 4, M–P). These findings jointly confirm that mutant TECs are more vulnerable to inflammatory stimuli, displaying a pronounced proinflammatory phenotype, which can be attenuated by dapagliflozin.

### Dapagliflozin Inhibits the STING Pathway in the Renal Cortex and TECs of *Col4a3*-Mutant Mice

**Figure 5:**
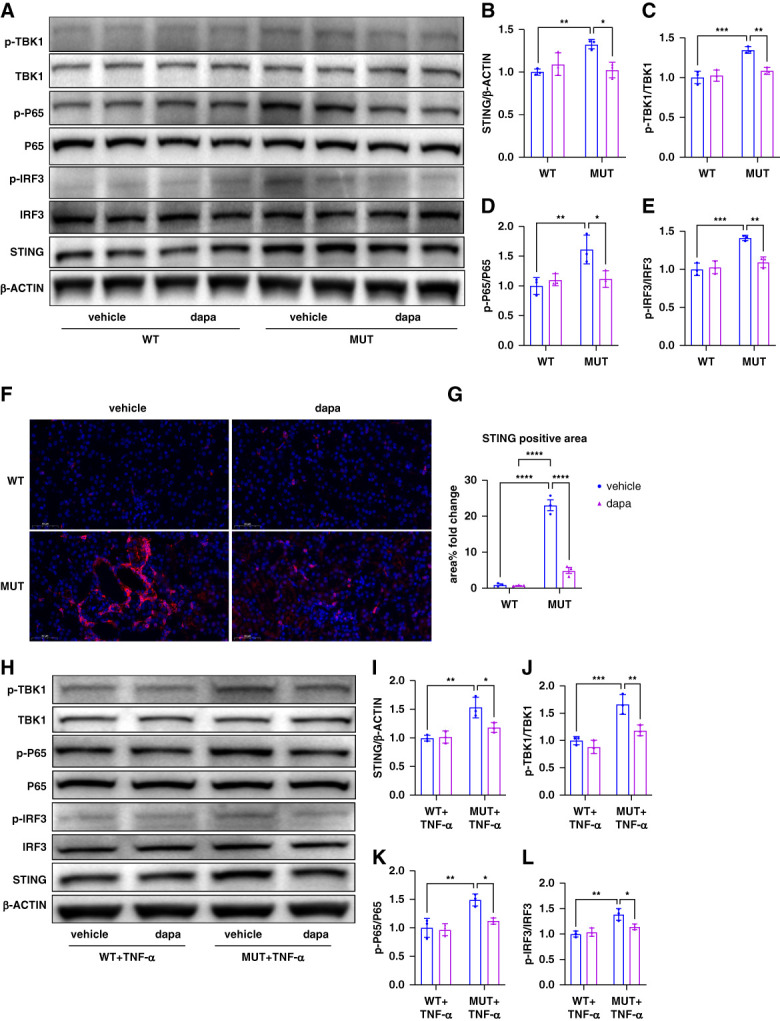
**Dapagliflozin inhibits STING pathway activation in the renal cortex and proximal TECs of *Col4a3* mutant mice.** (A–E) Representative western blot images and quantitation for the expression of p-TBK1, TBK1, p-P65, P65, p-IRF3, IRF3, and STING in renal cortex of mice in each group. (F) Immunofluorescence staining of STING expression in kidney section of mice in each group, bar:50 *μ*m. (G) Quantitative of positive area of STING staining in kidney of mouse kidney in each group. (H–L) Representative western blot images and quantitation for the expression of p-TBK1, TBK1, p-P65, P65, p-IRF3, IRF3, and STING of TECs under different conditions. *P < 0.05, *P < 0.01, ***P < 0.001, ****P < 0.0001. IRF3, IFN-regulatory factor 3; STING, stimulator of IFN genes.

The STING signaling pathway is a critical regulator of innate immunity and inflammation. To assess whether this pathway is activated in *Col4a3*-mutant kidneys, Western blot analysis of renal cortex tissue was performed. Mutant mice exhibited increased expression of STING, phosphorylated TBK1, NF-κB, and IRF3 (p-TBK1,p-P65, p-IRF3) compared with wild-type animals, whereas dapagliflozin treatment significantly reduced these levels (Figure 5, A–E). Immunofluorescence staining further confirmed that markedly increased STING expression in renal tubules of mutant mice which was reduced after dapagliflozin treatment (Figure 5, F and G). Consistent with *in vivo* findings, mutant TECs stimulated with TNF-*α* exhibited elevated STING, p-TBK1, p-P65, and p-IRF3 protein expression compared with wild-type cells, whereas these protein levels were reduced by dapagliflozin (Figure 5, H–L). Taken together, these findings suggest that the STING signaling pathway is activated in renal tubules of *Col4a3*-mutant mice and can be suppressed by dapagliflozin.

### Dapagliflozin Alleviates the Proinflammatory Phenotype of *Col4a3*-Mutant TECs Partially *via* STING Inhibition

**Figure 6:**
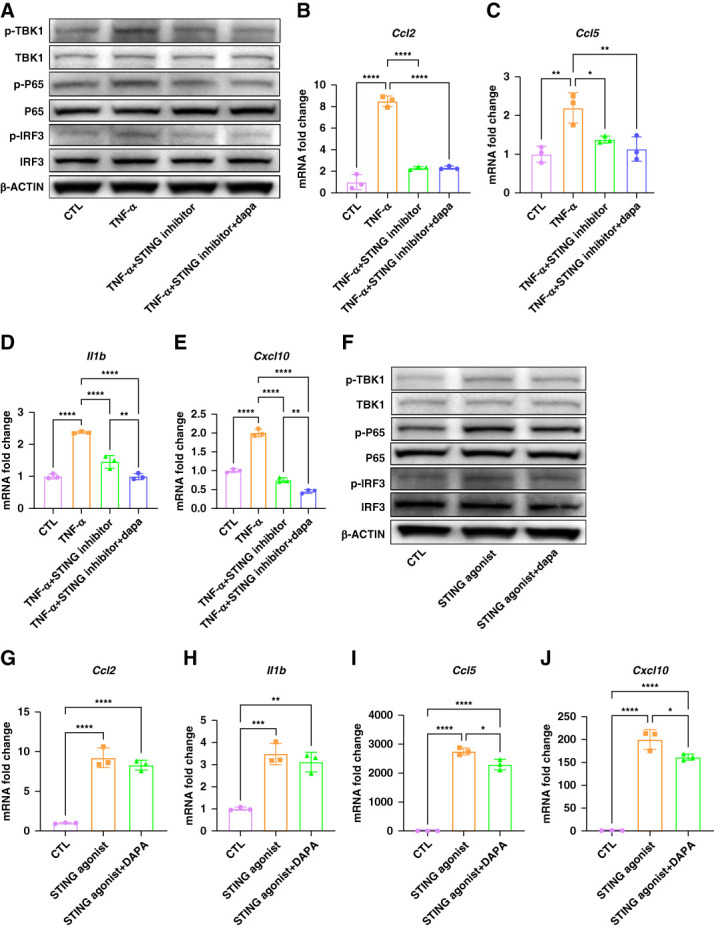
**Dapagliflozin alleviates the proinflammatory phenotype of mutant proximal TECs by inhibiting STING activation.** (A) Representative western blot images for the expression of p-TBK1, TBK1, p-P65, P65, p-IRF3, and IRF3 of mutant TECs under different conditions. (B–E) Transcription level of *Ccl2*, *Ccl5*, *Il1b*, and *Cxcl10* in mutant TECs under different stimulation. (F) Representative western blot images for the expression of p-TBK1, TBK1, p-P65, P65, p-IRF3, IRF3 of mutant TECs under different conditions. (G–J) Transcription level of *Ccl2*, *Il1b*, *Ccl5*, and *Cxcl10* in mutant TECs under different stimulation. *P < 0.05, *P < 0.01, ***P < 0.001, ****P < 0.0001.

To further clarify the mechanisms underlying anti-inflammatory of dapagliflozin, pharmacologic modulation of the STING pathway was performed. The STING inhibitor reduced the expression of p-TBK1, p-p65, and p-IRF3 in mutant tubules induced by TNF-*α* and dapagliflozin exerted a synergistic effect (Figure 6A). Meanwhile, treatment of mutant TECs with a STING inhibitor reduced TNF-*α* induced expression of *Ccl2*, *Ccl5*, *Cxcl10*, and *Il1b*. The combination of dapagliflozin and STING inhibition further decreased *Cxcl10* and *Il1b* transcript levels (Figure 6, B–E). Conversely, cotreatment with the STING agonist reversed dapagliflozin's effect on p-TBK1, p-p65, and p-IRF3 (Figure 6F). Stimulation with a STING agonist increased mRNA level of these cytokines in mutant TECs and attenuated inhibitory effects of dapagliflozin, with *Ccl2* and *Il1b* unchanged and *Ccl5* and *Cxcl10* partially reduced (Figure 6, G–J). These findings suggest that the SGLT2i exerts anti-inflammatory effects in mutant TECs partially through inhibition of STING activation.

## Discussion

SGLT2i therapy has been verified to slow disease progression and provide long-term benefits for people with CKD.^26^ In some small sample studies for patients with AS, SGLT2i showed a proteinuria-reducing effect in patients with ARAS or XLAS.^6–8^ A recent study conducted by Boeckhaus J *et al*. assessed 112 patients with AS and showed a significant reduction in ACR (>30%) during the first three follow-up visits after the initiation of SGLT2i therapy. However, they observed a mean loss of eGFR of 9±12 ml/min per 1.73 m^2^ nearly 1 year after baseline.^9^_._ Patients with AS are considered to be at a high risk of rapid progression of CKD. There is significant heterogeneity in the risk of progression among patients with different genotypes. The risk of progression to ESKD is approximately 20% to 25% in patients with autosomal dominant alport syndrome and female XLAS but nearly 100% in male XLAS and ARAS.^3^ Furthermore, patients with AS of different genotypes may exhibit variable responses to the angiotensin-targeting drugs.^27^ However, neither our investigation nor that of Boeckhaus J *et al*. included a placebo-controlled arm, making it difficult to compare eGFR changes with those in the absence of SGLT2i treatment.

The DAPA-CKD study pointed out that compared with non–type 2 diabetes mellitus CKD patients, dapagliflozin reduced urinary ACR to a larger extent in CKD patients with type 2 diabetes mellitus, while the effects on renal function and other clinical outcomes were similar in both groups, suggesting that the renoprotective effect of dapagliflozin on CKD may be mediated through other pathways unrelated to proteinuria reduction.^28^ Tubulointerstitial inflammation is not only the downstream event of glomerular lesions but also plays an independent role in the progression of renal function impairment. Dapagliflozin may further exert kidney protective effects by reducing renal interstitial inflammation.

*Col4a3* p.C1615Y-mutant mice were used as an AS animal model. This missense mutation corresponds to amino acid number 1616 in human.^29^ The Classical *Col4a3*^−/−^ mouse exhibits severe renal injury and dies at approximately 10–24 weeks of age depending on the genetic background.^30^ *Col4a3* p.C1615Y-mutant mice can better mimic the milder phenotype of patients with missense mutations.

In this study, H&E staining and F4/80 staining of kidney sections from mutant mice displayed increased immune cell(macrophage) infiltration. It was reported that inflammation is tightly associated with fibrosis and kidney disease progression.^31^ Chronic inflammation is an essential factor that aggravates AS nephropathy. In particular, macrophages are the most important inflammatory cells in the renal interstitium that promotes progression. Several experimental studies have demonstrated that relieving inflammation reduces collagen deposition, ameliorates renal function, and prolongs the survival of AS mice.^14,32–34^ We did not observe an improvement of renal function in the mutant mice, which may be related to the transient increase in blood creatinine commonly associated with the initial treatment with SGLT2i. In our animal experiments, dapagliflozin reduced immune cell infiltration and decreased the transcription levels of inflammatory cytokines. Our findings are consistent with a previous study that showed *Il1b* and *Ccl2* mRNA levels were increased in the renal cortex of *Col4a3*^*−/−*^ mice and were most significantly reduced by dapagliflozin and ramipril treatment.^35^ STING pathway activation recruits TBK1 and IκB kinase (IKK), which phosphorylate IRF3 and NF-κB inhibitor (IκBα), respectively, and thereby inducing the transcription of genes encoding type 1 IFN and proinflammatory cytokines.^36^ STING contributes to inflammatory activation in many kidney diseases, including AKI, autosomal dominant polycystic kidney disease, and diabetic nephropathy.^37–39^ Mitrofanova *et al*. demonstrated STING activation in the glomeruli of *Col4a3^−/−^* mice and elucidated its role in autophagic podocyte death.^40^ Our study revealed that STING is activated in renal tubules of *Col4a3*-mutant mice and drives a proinflammatory phenotype, contributing to kidney inflammation. Because the pathogenic mechanisms underlying tubular injury in AS remain incompletely understood, our findings extend the role of STING beyond the glomerulus, particularly with regard to tubular inflammation, and provide a comprehensive understanding of its potential involvement in AS progression.

In this study, we have not yet elucidated the molecular mechanism by which dapagliflozin inhibits the STING pathway, which requires further exploration. Cyclic GMP-AMP synthase recognizes mitochondrial DNA released upon organelle damage and then synthesizes cyclic GMP-AMP, which acts as a second messenger to activate the adaptor protein STING.^41,42^ Multiple studies have demonstrated that SGLT2i improve mitochondrial function, promote mitochondrial biogenesis, and reduce mitochondrial DNA leakage.^43,44^ Whether dapagliflozin suppresses STING-driven inflammatory activation by protecting renal tubular mitochondria therefore warrants further validation. As an endoplasmic reticulum (ER) adaptor protein, STING may be sensitive to ER stress and Ca^2+^ disturbances, which can lead to aberrant STING expression or trafficking. The ER Ca^2+^ sensor stromal interaction molecule 1 physically binds to STING and inhibits its translocation to the Golgi. The loss of stromal interaction molecule 1 triggers spontaneous STING activation.^45^ In addition, molecules, including coat protein complex II components and STING ER-exit protein are critical for STING's ER-to-Golgi trafficking,^46,47^ yet many details remain elusive. To date, no study has reported whether SGLT2i modulate STING trafficking by affecting these proteins. Recent studies have shown that canagliflozin preserves sarcoplasmic/ER Ca^2+^-ATPase activity, maintains cytosolic Ca^2+^ homeostasis, suppresses ER stress, reduces GRP78 translocation from the ER to the plasma membrane, and attenuates the epithelial-to-mesenchymal transition in proximal tubules of diabetic kidney disease.^48^ SGLT2i have been reported to alleviate ER stress in injured cardiomyocytes.^49^ Whether dapagliflozin mitigates STING activation by maintaining ER homeostasis remains to be experimentally verified.

Many studies showed that SGLT2i act as an anti-inflammatory drug and have been proposed as a useful strategy to treat diseases linked to inflammation.^50^ Several studies revealed dapagliflozin suppresses renal inflammation *via* NF-κB, necroinflammation, and NOD-like receptor family pyrin domain containing 3 inflammasome inhibition.^51–53^ We hypothesized multiple anti-inflammatory pathways may be affected by SGLT2i, and alternatively, the STING pathway may represent downstream mechanism, which deserves further investigation.

This study has several limitations. First, the clinical cohort was small and lacked a control group. Second, the upstream triggers and the precise mechanism underlying dapagliflozin's inhibition of STING remain unclear. In addition, we did not employ genetic silencing of STING, global or tubular-specific STING knockout models, or STING overexpression approaches to determine whether enforced activation abolishes the anti-inflammatory effects of dapagliflozin. The absence of these genetic gain- and loss-of-function strategies—including potential crosses with *Col4a3* missense mice—limits our ability to directly confirm that dapagliflozin's anti-inflammatory effects are mediated through STING suppression in renal TECs.

Overall, our data demonstrate that dapagliflozin could inhibit STING pathway activation in TECs of *Col4a3*-mutant mice, providing new mechanistic insights into the reno-protective role of SGLT2i.

## Supplementary Material

## Data Availability

All original data, including deidentified patient-level data or individual laboratory data measurements, are included in the manuscript and/or supplemental material.

## Declarative Statements

This study includes clinical experimentation and received Institutional Review Board or Ethics Committee approval. All patients provided written informed consent. This study includes clinical experimentation and complies with the Declaration of Helsinki. All animal experiments were conducted in accordance with the NIH Guide for the Care and Use of Laboratory Animals or an equivalent standard that meets or exceeds the ethical and welfare requirements outlined in the NIH Guide. All protocols were approved by the appropriate institutional animal care and use committee.

## Supplemental Material

Supplemental Table 1. Primer sequences used in this study.

Supplemental Table 2. Proteinuria and renal function of patients at baseline and last follow-up.

Supplemental Figure 1. KEGG pathway classification analysis in each group of renal cortex.
