# Supplementary material for: Dapagliflozin Reduces Kidney Inflammation in Alport Syndrome by Inhibiting the Stimulator of IFN Genes Pathway in Renal Tubular Epithelial Cells
Source: Kidney360. 2026 Jan 7;7(5):969–81. doi: 10.34067/KID.0000001099 (PMC13229431; doi:10.34067/KID.0000001099)
Supplement: Supplementary file 2 [file kidney360-7-0969-s002.pdf]

Supplemental Table 1: Primer sequences used in this study

Gene	Primer Forward	Primer Reverse
Gapdh	AAGAAGGTGGTGAAGCAGGCATC	CGGCATCGAAGGTGGAAGAGTG
Ccl2	CTGGAGCATCCACGTGTTGG	TCTTGAGCTTGGTGACAAAAACTAC
Ccl5	CCAATCTTGCAGTCGTGTTTGT	GGGGATTACTGAGTGGCATCC
Cxcl10	CCACGTGTTGAGATCATTGCC	GAGGCTCTCTGCTGTCCATC
Il1b	TGCCACCTTTTGACAGTGATG	TGATGTGCTGCTGCGAGATT
Tnf-a	CCCTCCAGAAAAGACACCATG	CACCCCGAAGTTCAGTAGACAG
Col1α1	CCCAGCCGCAAAGAGTCTAC	AGCATACCTCGGGTTTCCAC
Vimentin	AGACCAGAGATGGACAGGTGA	TTGCGCTCCTGAAAAACTGC

Supplemental Table 2: Proteinuria and renal function of patients at baseline and last follow up

No.	Baseline		Follow up time (month)	The last follow-up	
	24hUP (g)	eGFR (ml/min/1.73 m^2)		24hUP (g)	eGFR (ml/min/1.73 m^2)
1	1.09	102	41.8	1.56	92
2	1.79	35	34.9	1.63	32
3	0.30	65	20.3	0.20	67
4	1.90	112	12.0	0.70	106
5	0.79	56	40.6	0.67	51
6	1.06	79	34.4	0.46	82
7	1.66	40	30.1	0.73	39
8	1.82	37	12.3	0.76	41
9	1.20	94	19.4	1.18	92
10	1.21	107	15.2	0.71	101
11	2.3	93	20.6	1.58	88

12	1.26	102	12.7	0.82	107
13	1.32	115	18.5	0.87	119
14	1.93	52	20.7	1.09	36
15	1.03	35	23.6	0.65	32
16	2.83	107	18.3	1.79	86
17	4.11	66	17.3	2.65	63
18	3.19	59	12.8	2.80	59
19	1.54	71	14.6	2.16	58
20	1.54	128	26.8	1.80	112
21	2.89	66	21.7	2.30	64

A

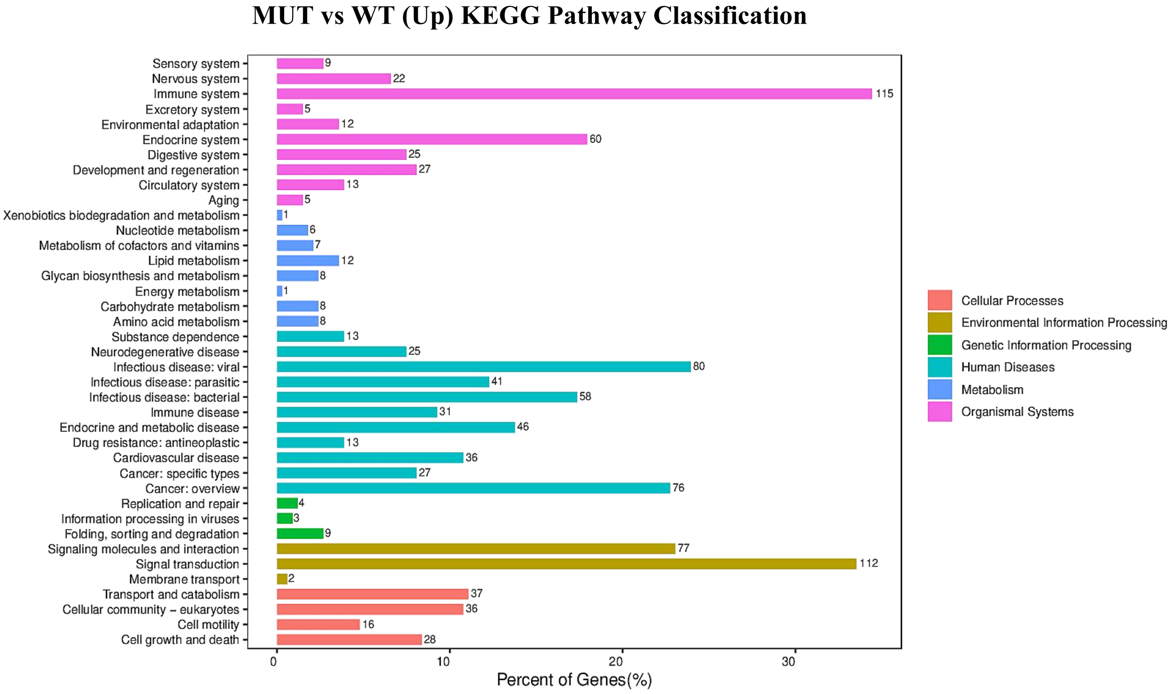

B

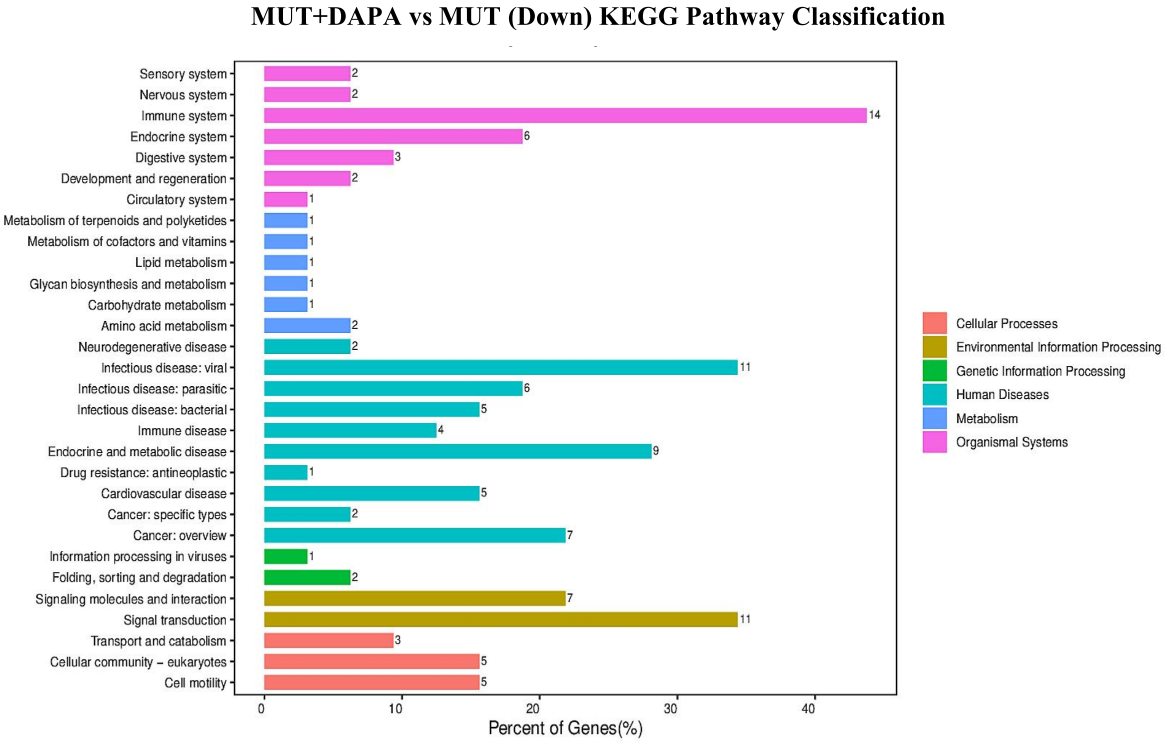

Supplemental Figure 1: KEGG pathway classification analysis in each group of renal cortex

A) KEGG enrichment analysis of DEGs of RNA transcriptomics in WT and MUT group (MUT vs WT)

B) KEGG enrichment analysis of DEGs of RNA transcriptomics in MUT and MUT+DAPA group

(MUT+DAPA vs MUT)
